# The Potential Mediating Role of Good Mental Health on the Relationship Between Low Physical Activity and High Screen Time with Executive Functions in Chilean Children and Adolescents

**DOI:** 10.3390/children12101402

**Published:** 2025-10-17

**Authors:** Felipe Caamaño-Navarrete, Carlos Arriagada-Hernández, Roberto Lagos-Hernández, Gerardo Fuentes-Vilugrón, Lorena Jara-Tomckowiack, Eduardo Sandoval-Obando, Guido Contreras-Díaz, Daniel Jerez-Mayorga, Claudio Hernández-Mosqueira, Pedro Delgado-Floody

**Affiliations:** 1 Physical Education Career, Universidad Autónoma de Chile, Temuco 4780000, Chile; felipe.caamano@uautonoma.cl (F.C.-N.); carlos.arriagada@uautonoma.cl (C.A.-H.); roberto.lagos@uautonoma.cl (R.L.-H.); gerardo.fuentes@uautonoma.cl (G.F.-V.); 2 Facultad de Educación, Universidad Católica de Temuco, Temuco 4780000, Chile; lorena.jarat@fmda.cl; 3 Escuela de Psicología, Instituto Iberoamericano Para el Desarrollo Sostenible, Facultad de Ciencias Sociales y Humanas, Universidad Autónoma de Chile, Temuco 4800916, Chile; eduardo.sandoval@uautonoma.cl; 4 Escuela de Kinesiología, Facultad de Ciencias de la Rehabilitación y Calidad de Vida, Universidad San Sebastián, Lago Panguipulli 1390, Puerto Montt 5501842, Chile; guido.contreras@uss.cl; 5 Department of Physical Education and Sports, Faculty of Sport Sciences, Sport and Health University Research Institute (iMUDS), University of Granada, 18012 Granada, Spain; djerezmayorga@ugr.es; 6 Exercise and Rehabilitation Sciences Institute, Faculty of Rehabilitation Sciences, Universidad Andres Bello, Santiago 7591538, Chile; 7 Departamento de Ciencias de la Educación, Universidad del Bio-Bio, Chillán 3800708, Chile; chernandez@ubiobio.cl; 8 Department of Physical Education, Sport and Recreation, Universidad de La Frontera, Temuco 4811230, Chile

**Keywords:** cognition, mental health, lifestyle, childhood and adolescence

## Abstract

**Highlights:**

**What are the main findings?**

- Good mental health partially mediates the link between an unhealthy lifestyle and executive functions.
- Low physical activity and high screen time were inversely related to attention, inhibition, cognitive flexibility, and working memory.

**What is the implication of the main finding?**

- Promoting active lifestyles and mental well-being from early ages may enhance cognitive development.
- Schools and families are key in integrated actions for physical activity, mental health, and screen use.

**Abstract:**

Background: Childhood and adolescence are increasingly recognized as life stages that pose specific challenges for treating and promoting mental health and cognitive development. Objective: The objective of the present study was to determine the potential mediating role of good mental health in the association between an unhealthy lifestyle (i.e., low physical activity (PA) and high screen time (ST)) with executive functions (EFs) (i.e., attention, inhibition, cognitive flexibility, and working memory) in children and adolescents. Methods: A cross-sectional investigation with 625 students aged 10–17 years participated. The Krece Plus questionnaire (lifestyle, PA, and ST), Depression Anxiety and Stress Scale (DASS-21, metal health), and CogniFit (EFs) were used in the present study. Results: Good mental health presented a partial mediating role in the relationship between a bad lifestyle and EFs. Likewise, a bad lifestyle was linked inversely to attention (β −37.45, *p* = 0.002), the executive function of cognitive flexibility (β −85.91, *p* < 0.001), inhibition (β −60.16, *p* < 0.001), and working memory (β −75.73, *p* < 0.001). Conclusions: Good mental health acts as a relevant mediator in child and adolescent cognitive development. These results reinforce the need to promote active and healthy lifestyles, as well as strategies that promote psychological wellbeing from an early age. Schools and families play an important role as protective agents and promoters of integral development; it is therefore recommended to implement intervention programmes that strategically address the physical activity, mental health, and digital habits of this population.

## 1. Introduction

Childhood and adolescence are increasingly recognized as life stages that pose specific challenges for treating and promoting mental health and cognitive development. The scientific literature has explicitly highlighted the importance of mental health in the human being [1]. Likewise, childhood and adolescence are critical periods for the development of psychological wellbeing [2]. Establishing good levels at an early age is therefore critical due to the implications for mental wellbeing in adulthood [3], and so, estimations and analyses of mental health in schools have garnered increased interest [4]. The evidence highlights that young people’s mental health is a global public health challenge (i.e., problems affect 10–20 per cent of children and adolescents worldwide) [5]. For example, since the mid-twentieth century, mental illness has positioned itself as one of the most relevant burdens for the health systems of countries, particularly among adolescents [6].

Mental health components could have negative effects on cognitive, emotional, and social development [7]. The stress faced by the population in this age group has an impact on child and youth wellbeing [8]. Research has estimated a high prevalence of mental disorders with an average onset before the age of 14 [9]. In Chile, high rates of anxiety, depression, and stress have been identified in this population; these have been associated with multifactorial situations [10]. Mental health not only influences individual wellbeing but also academic performance [11] and social relations, highlighting its relevance as a key component of human development [12], which includes, among other capacities, executive functions [13] such as attention, inhibition, cognitive flexibility, and working memory [14], these being essential skills for learning, emotional regulation, and decision-making. In this study, we adopt a two-dimensional model of mental health, which recognizes both the absence of negative emotional states (depression, anxiety, and stress) and the presence of positive wellbeing. This approach was chosen because it provides a more comprehensive and consistent framework for understanding child and adolescent development.

EFs have been defined as higher-order cognitive skills oriented towards the achievement of objectives (intentional behaviour) based on the performance of complex tasks [15,16]. They allow a person to regulate their attention span, keep objectives and information in mind, avoid automated responses, and reflect on the consequences of different behaviours [17]. In addition, they are vital mental processes in novel situations that require the rapid and flexible adjustment of behaviour in response to changing environmental demands [18]. There is consensus in establishing the three dimensions related to EFs: inhibition and control of interference (i.e., attention and concentration), working memory, and cognitive flexibility [19]. In addition, it has been established that developing EFs could have beneficial effects on different dimensions of human beings’ lives [13]. High cognitive development during early stages has been associated with improved mental health in adulthood [20].

Traditionally, the scientific literature has reported that a healthy lifestyle is positively related to variables associated with health and a decrease in the risk of death [21]. However, several studies have broadened the view, generating strong evidence that a healthy lifestyle is positively associated with EFs and mental health [22,23]. Consistent with this, it has been indicated that regular participation in physical activity (PA) programmes is positively related to working memory, inhibition, problem solving, and cognitive skills [24]. On the other hand, one study indicated that high screen time (ST) had a negative impact on cognition [25]. Therefore, the components of a healthy lifestyle must be diagnosed according to their relationship with different variables of interest for the school context. In line with the above, it has been indicated that improving one’s lifestyle could be an effective tool to positively impact mental health and EFs [26,27].

Studies have shown that children’s PA favourably affects mental health in youth [28,29] and acts as a mediator that promotes wellbeing and generates a decrease in anxiety [30], suggesting that interventions of this nature could have positive effects, including on symptoms of attention deficit hyperactivity disorder [31]. Despite this background, the impact of PA and its specific role as a mediator in mental health and lifestyle has not been systematically explored in Chile [32]. In relation to an unhealthy lifestyle, characterized by low PA and high ST [33], PA has been considered a critical aspect of mental health in child and youth populations [34]. Although some of the instruments applied have been previously validated in Chile, it is important to consider that cultural, educational, and socioeconomic factors in this context may generate distinctive patterns that differ from international evidence. Therefore, examining these associations locally provides a more nuanced understanding of child and adolescent development in Chile. In Chile, data reveal that active adolescents report positive wellbeing [27]; although the harmful effect of excessive screen use [35] is not necessarily compensated by PA [32], it is positively associated with working memory and cognitive flexibility and a better quality of life [27], which is also related to eating habits [36]. Evidence indicates that participation in team sports favours EFs [37] and that vigorous PA and good sleep quality improve working memory and cognitive flexibility [38]. Thus, these physical skills are associated with better cognition and represent mediators directly related to executive functions [39], while their absence is related to consequences for sleep, quality of life, and cognitive development. In this study, we conceptualize mental health as an affective–emotional state (levels of depression symptoms, anxiety, and stress) that can either facilitate or hinder the optimal functioning of EFs. We therefore adopt a mediating (not moderating) framework: lifestyle may influence EFs performance indirectly through its impact on mental health rather than changing the strength of the lifestyle–EF association across levels of mental health.

This study contributes to the growing body of scientific evidence that supports the design of interventions aimed at improving mental health and EFs in students, as well as how good mental health can mediate the link between lifestyle and cognition. The mediation of mental health and its link with lifestyle and EFs have been explored in international contexts [40], suggesting that PA could have favourable effects, interceding against EFs [41], with a favourable influence on academic performance, self-efficacy, and emotional regulation. Therefore, observing the potential mediating role in mental health in association with lifestyle and EFs, based on Chilean children and adolescents, constitutes a substantive contribution to the understanding of this phenomenon. Given the cross-sectional nature of the research, the study hypothesizes that an unhealthy lifestyle is negatively associated with EFs, and better mental health mediates these associations. Considering this background, the aim of the present study was to determine the potential mediating role of good mental health in the association between an unhealthy lifestyle (i.e., low physical activity and high screen time) with executive functions (i.e., attention, inhibition, cognitive flexibility, and working memory) in children and adolescents.

## 2. Materials and Methods

### 2.1. Participants and Study Design

The present study employed a quantitative, cross-sectional, and descriptive-associative design. A total of 625 Chilean youth aged 10 to 17 years (mean age 13.68 ± 1.65 years) from various schools in Temuco, Chile, participated in the present investigation. A total of twenty-three students were excluded. The sample was intentional and non-probabilistic. In this regard, the main limitation of results from non-probabilistic data is their uncertain and limited generalizability to the population of interest [42]

The sample size was calculated considering the following factors: (1) enrolment of students in educational institutions appropriate for their age group (10–17 years); (2) a significance level of 5 percent; (3) an absolute precision of 5 percent; (4) a statistical power of 95 percent; (5) the statistical test (*t*-test); (6) the number of measurements (x1); and (7) an effect size of 0.2. Based on these parameters, and accounting for an expected response rate of 80 percent, a sample size of 400 participants aged 10 to 17 years was determined.

The inclusion criteria were as follows: (i) subjects had to be enrolled in school and (ii) be aged between 10 and 17 years. The exclusion criteria included: (i) any medical contraindications that would prevent normal performance in the assessments and (ii) absence during the evaluations.

The study adhered to the principles outlined in the 2013 Declaration of Helsinki and was approved by the Ethics Committee of the Universidad Autónoma de Chile (approval number: CEC 13-25). Participation in the research required signed consent from the students themselves, as well as informed consent from their parents.

### 2.2. Main Outcomes

#### 2.2.1. Lifestyle

PA patterns were determined with the Krece Plus test [43]. Krece Plus is a quick questionnaire that classifies lifestyle based on the average number of hours spent watching television or playing video games per day (ST) and the hours of PA after school per week. The classification is made according to the number of hours spent on each item. The total points are added, and the person’s lifestyle is classified as good (men ≥ 9, women ≥ 8), regular (men 6–8, women 5–7), or bad (men ≤ 5, women ≤ 4), according to the lifestyle score. The Krece Plus instrument has been used previously with Chilean students [44] and has shown moderate internal consistency (Cronbach’s alpha 0.79) [45].

#### 2.2.2. Executive Function

To evaluate EFs, including inhibition, working memory, cognitive flexibility, and attention, the CogniFit neurocognitive assessment battery (San Francisco, CA, USA) was used [46]. This 40 min assessment provides both a general cognitive score and specific scores for EFs. The CogniFit battery has been reported to exhibit good reliability and has been successfully used with school-aged subjects [47]. Moreover, CogniFit has been used previously with Chilean schoolchildren [36]. Cognifit^©^ is used individually to measure the cognitive functioning of all participants. This cognitive profile has demonstrated high reliability, consistency, and stability in previous studies [48]. CogniFit has shown good internal consistency (Cronbach’s alpha 0.85–0.88) [49].

#### 2.2.3. Mental Health

The abbreviated version of the Depression Anxiety Stress Scale (DASS-21) was used to determine mental health problems [50]. This three-dimensional self-report scale was designed to evaluate the presence and intensity of emotional states or symptoms of depression, anxiety, and stress [51,52,53]. The questionnaire is composed of 21 questions divided into three subscales: depression (feelings of dysphoria, hopelessness, devaluation of life, self-hatred, lack of interest, and anhedonia); anxiety (evaluates experiences of physiological arousal, situational anxiety, and general anxiety); and stress (evaluates levels of irritability, feelings of overwhelm, problems relaxing, and difficulty controlling excessive thinking). Questions are scored on a scale from 0 (“It doesn’t describe anything that happened to me or that I felt during the week”) to 3 points (“Yes, this happened to me a lot, or most of the time”) according to the degree of intensity in the last week. The sum of the questions is therefore within the range of 0 to 21 points. Lower scores corresponded to reduced symptoms. A score > 5 points was the cut-off point for depression and stress symptoms. A score > 4 points was the cut-off point for anxiety symptoms [54]. This instrument has the advantages of being a self-report scale, brief, easy to administer and answer, and easy to interpret [55]. These questionnaires have been used with Chilean students [56] and presented adequate psychometric properties [55]. DASS-21 has shown good internal consistency (Cronbach’s alpha > 0.80) in adolescent subjects [57].

The research team responsible for evaluating the adolescent participants was trained in the study protocols and assessment procedures prior to data collection. Subjects completed the questionnaires and evaluations individually under the supervision of researchers who were available to address any questions or concerns.

### 2.3. Statistical Analysis

The data are presented as means and standard deviations (SD). Normality assumptions and homoscedasticity were analyzed using the Kolmogorov–Smirnov test and the Levene test, respectively. *t*-test was used to identify differences between sexes (girls vs. boys); to compare differences in lifestyle, analysis of variance (ANOVA) was used; meanwhile, the Bonferroni post hoc test was applied to test differences among groups according to lifestyle (good vs. regular vs. bad lifestyle). Overall, the alpha level was set at *p* < 0.05 for statistical significance.

To examine the association between lifestyle and EFs, lineal regression models were applied and adjusted for age (Model 2) and sex (Model 3). The results are reported as Unstandardized Coefficients of beta coefficients (β) and 95% confidence interval [CI]). In addition, the Standardized Coefficients of beta and standard error (SE) were added.

Regression analyses were performed to verify the effect of the mediating variable good mental health (including anxiety, stress, and depression symptoms) (M), considering bad lifestyle (i.e., low PA and high ST) as the independent variable (X) and EFs as the dependent variables (Y). Within the analysis, the total effect (c), direct effect (c′), and indirect effect (a*b, IE) were calculated for the samples, as well as the 95 percent CI using the macro/interface PROCESS v. 3.3 for SPSS v. 23 and the bootstrapping method with a resampling rate of 5000 [58]. The percentage of mediation was estimated as a proportion between the direct and the total effect 1 − (c′/c). All the statistical analyses were performed using SPSS statistical software version 23.0 (SPSS^TM^ Inc., Chicago, USA). The alpha level was set at *p* < 0.05 for statistical significance.

## 3. Results

Table 1 shows the comparison of study variables according to sex. Boys reported higher levels of PA than girls (*p* < 0.001). The girls presented worse symptoms (*p* < 0.001) in anxiety, depression, and stress. Compared with girls, boys reported higher scores in cognitive flexibility (*p* < 0.001).

Table 2 presents the results for the frequency of mental health problems according to sex. There were significant differences in mental health variables according to sex. The lifestyle categories presented no significant differences (*p* > 0.05).

Table 3 shows the association between EFs and a bad lifestyle. Bad lifestyle was inversely linked to attention (β −37.45, *p* = 0.002); when the association was adjusted by sex and age, the significant association remained (Model 3). The function executive of cognitive flexibility in a model adjusted by sex and age (Model 3) reported an inverse association with bad lifestyle (β −85.91, *p* < 0.001). The inhibition variable presented an inverse association with bad lifestyle, adjusted for sex and age (β −60.16, *p* < 0.001, Model 3). Finally, working memory was inversely linked to bad lifestyle (β −75.73, *p* < 0.001).

**Figure 1:**
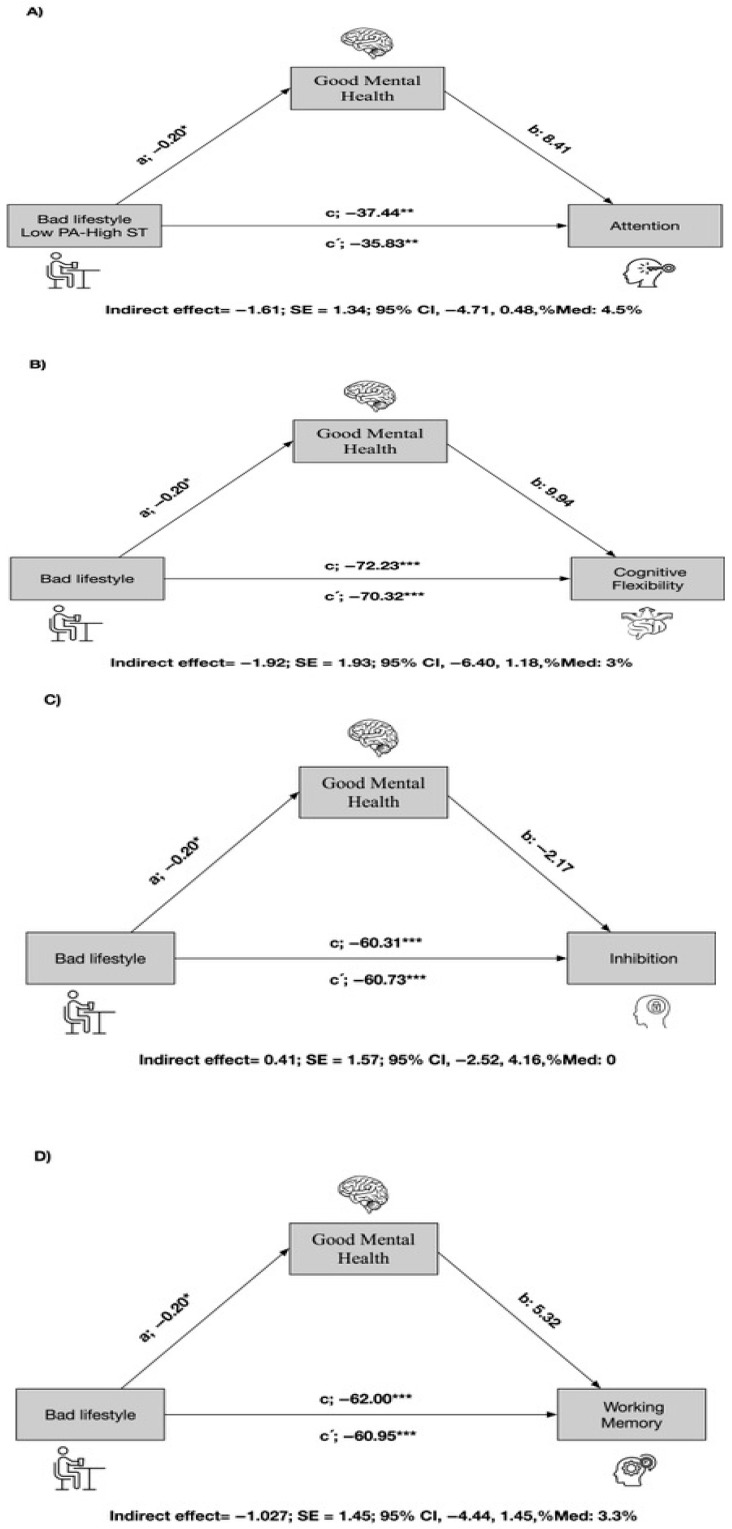
The mediation analysis for the total sample. * *p* < 0.05, ** *p* < 0.001, *** *p* < 0.0001.

The mediation analysis results for the total sample (n = 625 children and adolescents) are shown in Figure 1. Good mental health emerged as a mediating variable in the relationship between a bad lifestyle and EFs. In the first regression step (a), a bad lifestyle was inversely related to mental health. In the second step (c’), the regression coefficient of a bad lifestyle in attention was also significant. In the third step, the potential mediator of mental health was positively related to the dependent variable (b); when both bad lifestyle and mental health were included in the model (c), the regression coefficient remained statistically significant (*p* < 0.001). Finally, the indirect effect confirmed that mental health was a partial mediator of attention (indirect effect −1.61; SE 1.34; 95% CI −4.71–0.48; and Med 4.5%) (see Figure 1A). Similarly, the potential mediator of mental health was positively related to the dependent variables (b) of cognitive flexibility (Figure 1B) and to working memory (Figure 1D). Finally, the indirect effect confirmed that mental health was a partial mediator of cognitive flexibility (indirect effect −1.92; SE 1.93; 95% CI −6.40–1.18; and Med 3%) and working memory (indirect effect −1.027; SE 1.45; 95% CI −4.44–1.45; and Med 3.3%). Mental health presented no mediation in the association between a bad lifestyle and the EF of flexibility.

## 4. Discussion

The objective of this study was to determine the potential mediating role in the association between an unhealthy lifestyle (i.e., low PA and high ST) and EFs (i.e., attention, inhibition, cognitive flexibility, and working memory) in children and adolescents. The main results of this study were as follows: (i) mental health presented as a mediator role in the relationship between a bad lifestyle (i.e., high ST and low PA) and EFs; (ii) bad lifestyle was inversely associated with all EFs; and (iii) girls reported lower levels of cognitive flexibility and lower mental health than boys.

In the present study, mental health presented a mediator role in the relationship between an unhealthy lifestyle (i.e., high ST and low PA) with the EFs of (i) attention, (ii) cognitive flexibility, and (iii) working memory. Although we regard executive function as an overarching concept, its various elements react in distinct ways to lifestyle factors and mental health influences. Aspects like attention and working memory seem more immediately affected by stress and the management of emotions, which accounts for the anticipated more pronounced mediating role of mental health in those specific areas. Another study, which had the objective of determining the potential mediating role of subjective wellbeing (i.e., health-related quality of life, HRQoL) in the association between ST and EFs, showed that HRQoL partially mediated the relationship between ST and EFs in schoolchildren, including attention (5%), inhibition (3.18%), working memory (3.82%), and cognitive flexibility (5.3%) [27]. This finding is supported by recent research such as that by Caamaño-Navarrete et al. [36], in which it was observed that an unhealthy lifestyle was inversely associated with negative mental health in Chilean schoolchildren. Halse et al. [59] found, in a longitudinal analysis, that poorer mental health (i.e., increased depression, anxiety, ADHD, and ODD/CD) may negatively impact executive functioning among Norwegian children. A systematic review with metanalysis highlighted that adolescents with mental health issues such as depression showed lower performance on cognitive tasks [60]. Research has proven that a better childhood cognitive ability is linked to fewer mental health problems (i.e., symptoms of anxiety and depression) in adulthood in women from the British 1946 birth cohort [61]. It has been shown that depressive symptoms mediate the association between a healthy lifestyle and cognitive function [62]. Another study found that students with a better cognitive ability had higher levels of mental health (i.e., higher levels of global self-esteem), fewer behaviour problems, and reduced hyperactivity and inattention problems [63]. The fact that mental health and EFs have a complex interactive relationship was obtained from exhaustive studies [61]. These findings underline that mental health is an important factor to consider in understanding the relationship between lifestyle and EFs; therefore, improving mental health could buffer the negative effects of low PA and high ST on the cognition of children and youths.

Likewise, an unhealthy lifestyle was inversely associated with all EFs: (i) attention, (ii) cognitive flexibility, (iii) inhibition, and (iii) working memory. This aligns with prior research, such as the study on lifestyle and cognition among Chilean schoolchildren that found that children’s lifestyles (i.e., high ST and low MD adherence) were negatively associated with EFs [44]. Complementary to the above, previous evidence has shown that a bad lifestyle could have a negative effect on attention [64]. Another study has shown that high ST contributes to adverse cognitive, EF, and behaviour outcomes [65]. Similarly, a recent study indicated that excessive ST was negatively related to EFs in children [66]. Furthermore, evidence indicates that ST has adverse consequences on brain structure and intelligence [67]. Similarly, the study by Cadenas-Sánchez et al. [68] highlighted that sedentary time was negatively linked to the shape of subcortical brain structures, which in turn was related to intelligence in schoolchildren. Complementary to the above, after a two-year follow-up study, Chen et al. [69] suggested that a negative long-term impact of increased daily ST on children’s neuropsychological development. The study conducted by Lewin et al. [38] on schoolchildren demonstrated that ST was linked to reduced brain activation during an inhibitory control task. Complementary to the above, Jirout et al. [70] reported that modifiable lifestyle factors such as diet quality, PA, and sleep patterns are important for learning skills in school. Indeed, good food habits and higher PA are good predictors of EFs despite the inverse link between obesity and EFs among Malaysian adolescents [71].

Additionally, another study highlighted that a lifestyle characterized by a lack of PA could have detrimental effects on cognitive functioning [72]. The study conducted by Daniele et al. [73] indicated that physical inactivity progressively led to reduced peripheral and brain vasculature. Meanwhile, the study by McMath et al. [74] reported that meeting guidelines for PA and ST was related to greater EFs. Our findings are supported by research by Zeng et al. [75], in which it was observed that low ST and high PA were positively associated with the development of EFs in children. The systematic review and metanalysis by Álvarez-Bueno et al. [76] demonstrated that PA benefits several domains of cognition and metacognition in youths. Moreover, it has been indicated that PA in youth is positively related with cognitive benefits [77]. This aligns with prior research highlighting the crucial role of PA interventions and programmes on EFs at the school stage [75]. Several researchers have affirmed that PA can influence brain function through the modulatory effects of brain-derived neurotrophic factor (BDNF) and hormones and metabolites of the muscle–brain axis such as irisin, lactate, cathepsin B, kynurenine, and insulin-like growth factor-1 (IGF-1) [78,79], and school is among the most relevant factors to promote PA in children.

Girls reported lower levels of cognitive flexibility. Another study [80] also reported sex differences in EFs (i.e., inhibition task). Additionally, a study carried out by Gaillard et al. [81] claimed that there is evidence for sex differences in the neural networks underlying all tasks of executive control. Another study among children reported sex differences in neutral reaction time (RT) in a flanker task and go accuracy in the go/no-go task [82]. A metanalysis also provided evidence for gender-specific networks in working memory, whereby females consistently activate more limbic (e.g., amygdala and hippocampus) and prefrontal structures (e.g., right inferior frontal gyrus) [83]. On the other hand, a systematic review and metanalysis reported that males and females did not differ in performance in any of the three EF domains [84].

Moreover, we found that girls presented lower mental health than boys. Sex differences in adolescents’ mental health problems have been extensively reported. A longitudinal study reported that young people, particularly girls, are at increased risk of mental health problems between the ages of 11 and 14 [85]. A study on mental health in adolescents demonstrated that girls had higher levels of anxiety and depression symptomatology than boys [86]. Furthermore, evidence indicates that there appear to be sex and gender differences in mental health prevalence and risk factors [87]. In addition, it has been shown that mental health, cognitive development, and social background are linked to educational success; therefore, promoting positive mental health can improve children’s learning outcomes [88].

The findings of this study have significant implications for the development of targeted interventions aimed at promoting healthy lifestyles among Chilean students. Educational programmes focusing on PA are crucial. Furthermore, schools should consider implementing policies that support healthy behaviours, such as providing access to affordable and nutritious meals, creating opportunities for PA, and promoting awareness of the importance of healthy lifestyle. Likewise, scientific evidence highlights the importance of stimulating the development of healthy habits in the school context. A systematic review highlighted the importance of having a healthy lifestyle due to the benefits it provides at the mental level and for subjective wellbeing [89]. Therefore, it is important to continue researching the relationship between lifestyle and different variables of importance in the school context such as EFs and mental health. In a future study, we plan to implement short, high-intensity physical activity breaks in classrooms to determine their impact on high-intensity, short-duration cognitive and mental health variables.

This study has some limitations that should be considered when interpreting the results. Firstly, the cross-sectional design of this study prevents establishing causal relationships between lifestyle, mental health, and EFs, limiting inferences to correlational associations. Secondly, a non-probabilistic and intentional sample was used, which restricts the generalization of the findings to other child and adolescent populations. Thirdly, another limitation is the absence of contextual variables, such as the socioeconomic level or family environment. Future research should also consider incorporating objective measures of PA and ST.

## 5. Conclusions

In conclusion, this study highlights that an unhealthy lifestyle, characterized by unhealthy lifestyle habits such as low physical activity and high screen time, is inversely associated with the executive functions of Chilean children and adolescents, especially in attention, cognitive flexibility, and working memory. In addition, good mental health acts as a relevant mediator in child and adolescent cognitive development. These results reinforce the need to promote an active and healthy lifestyle along with strategies that promote psychological wellbeing from an early age. Schools and families play an important role as protective agents and promoters of integral development, so it is recommended to implement intervention programmes that strategically address the physical activity, mental health, and digital habits of this population.

## Figures and Tables

**Table 1:** Characteristics of the study sample and comparison by sex.

	Boys (n = 306)	Girls (n = 319)	Total (n = 625)	*p*-Value	F-Value
Age (y)	13.20 ± 1.75	13.23 ± 1.91	13.22 ± 1.83	0.859	0.03
Screen time (h/day)	3.40 ± 1.62	3.041 ± 1.44	3.22 ± 1.54	0.300	8.72
Physical activity (h/week)	2.12 ± 1.17	1.73 ± 1.10	1.92 ± 1.15	*p* < 0.001	17.88
Lifestyle	3.80 ± 1.90	3.75 ± 1.81	3.78 ± 1.85	0.736	0.11
Executive functions					
Attention (score)	435.59 ± 148.64	405.42 ± 155.72	419.80 ± 153.00	0.017	5.68
Cognitive flexibility (score)	385.48 ± 245.60	330.18 ± 240.03	356.59 ± 244.07	0.006	7.51
Inhibition	284.35 ± 222.88	301.06 ± 243.98	293.08 ± 234.09	0.391	0.74
Working Memory(score)	208.48 ± 211.79	195.66 ± 195.46	201.78 ± 203.34	0.449	0.57
Mental health					
Anxiety	6.32 ± 4.99	9.37 ± 5.81	7.88 ± 5.63	*p* < 0.001	49.57
Depression	6.70 ± 4.92	9.27 ± 5.77	8.01 ± 5.52	*p* < 0.001	35.94
Stress	8.40 ± 4.86	10.80 ± 5.28	9.62 ± 5.21	*p* < 0.001	34.90

The values are presented as mean ± SD; a *p*-value < 0.05 was considered statistically significant.

**Table 2:** Frequency of mental health problems according to sex.

		Boys		Girls		Total		
		n	%	n	%	n	%	
Anxiety	Absence	113	36.9%	63	19.7%	176	28.2%	*p* < 0.001
	Mild	25	8.2%	12	3.8%	37	5.9%	
	Moderate	51	16.7%	56	17.6%	107	17.1%	
	Severe	37	12.1%	35	11.0%	72	11.5%	
	Extremely severe	80	26.1%	153	48.0%	233	37.3%	
	Total	306	100.0%	319	100.0%	625	100.0%	
Depression	Absence	120	39.2%	76	23.8%	196	31.4%	*p* < 0.001
	Mild	50	16.3%	33	10.3%	83	13.3%	
	Moderate	64	20.9%	80	25.1%	144	23.0%	
	Severe	38	12.4%	52	16.3%	90	14.4%	
	Extremely severe	34	11.1%	78	24.5%	112	17.9%	
	Total	306	100.0%	319	100.0%	625	100.0%	
Stress	Absence	138	45.1%	91	28.5%	229	36.6%	*p* < 0.001
	Mild	45	14.7%	31	9.7%	76	12.2%	
	Moderate	54	17.6%	63	19.7%	117	18.7%	
	Severe	54	17.6%	84	26.3%	138	22.1%	
	Extremely severe	15	4.9%	50	15.7%	65	10.4%	
	Total	306	100.0%	319	100.0%	625	100.0%	
Lifestyle	Bad lifestyle	193	65.2%	208	66.7%	401	66.0%	*p* = 0.434
	Regular lifestyle	78	26.4%	86	27.6%	164	27.0%	
	Good lifestyle	25	8.4%	18	5.8%	43	7.1%	
	Total	296	100.0%	312	100.0%	608	100.0%	

Data are presented as n and proportions (%). A *p*-value of less than 0.05 was considered statistically significant.

**Table 3:** Association between negative lifestyle with executive functions.

		β	95% CI		Beta	SE	*p*-Value
	Model		Attention				
Negative Lifestyle	1	−37.45	−61.12	−13.78	−0.13	12.05	0.002
	2	−37.74	−61.29	−14.19	−0.13	11.99	0.002
	3	−44.23	−67.93	−20.54	−0.15	12.06	*p* < 0.001
Cognitive Flexibility							
Negative Lifestyle	1	−72.23	−109.72	−34.75	−0.16	19.08	*p* < 0.001
	2	−72.79	−110.05	−35.54	−0.16	18.97	*p* < 0.001
	3	−85.91	−123.03	−48.78	−0.19	18.90	*p* < 0.001
Inhibition							
Negative Lifestyle	1	−60.32	−96.18	−24.45	−0.14	18.26	0.001
	2	−60.16	−96.04	−24.28	−0.14	18.27	0.001
	3	−65.77	−102.26	−29.29	−0.15	18.57	*p* < 0.001
Working Memory							
Negative Lifestyle	1	−61.98	−93.19	−30.77	−0.16	15.89	*p* < 0.001
	2	−62.15	−93.36	−30.94	−0.16	15.89	*p* < 0.001
	3	−75.73	−106.61	−44.85	−0.20	15.72	*p* < 0.001

Model non-adjusted (Model 1); model adjusted by age (Model 2); and model adjusted by age and sex (Model 3). *p*-value < 0.05 was considered statistically significant.

## Data Availability

The original contributions presented in this study are included in the article. Further inquiries can be directed to the corresponding author.
